# Efficacy of Ultrasonic Cleaning Products With Various Disinfection Chemistries on Dental Instruments Contaminated With Bioburden

**DOI:** 10.1016/j.identj.2025.02.009

**Published:** 2025-03-25

**Authors:** Chaminda Jayampath Seneviratne, Sadaf Aiman Khan, Jessica Zachar, Zhihe Yang, Ramya Kiran, Laurence J. Walsh

**Affiliations:** School of Dentistry, The University of Queensland, Brisbane, Australia

**Keywords:** Ultrasonic cleaning products, Dental instruments, Bioburden, Infection control

## Abstract

**Objectives:**

The effective cleaning of reusable dental instruments that removes organic bioburden is a crucial process in infection prevention and control in dental clinics. Despite widespread use, the parameters affecting the efficacy of ultrasonic cleaning products with different chemistries remain underexplored. In the present study, we comprehensively evaluated the cleaning efficacy of commonly available cleaning detergent products against organic bioburden on dental instruments.

**Methods:**

Thirteen commercially available cleaning detergent products were assessed using Browne STF Load Check Indicators under both static and ultrasonic cleaning conditions at room temperature (25°C) and warm temperature (40°C). Experiments evaluated the effect of product concentration and contact time (1, 5, 10, and 30 minutes), and the economic impact of cleaning detergent dilution. Cleaning efficacy was also tested against artificially soiled dental instruments using ProReveal fluorescence technology.

**Results:**

Significant variability in cleaning efficacy among test products was observed. Optizyme Ultra (6 mL/L), Asepti Multizyme (8 mL/L), and Getinge Enzymatic Plus (20 mL/L) demonstrated superior cleaning performance, particularly when used in ultrasonic cleaners at 40°C. In general, enzymatic products consistently outperformed nonenzymatic products for the removal of organic bioburden. Products performed better with ultrasonic agitation than under static conditions, and optimal results were obtained after 10 minutes exposure time at 40°C.

**Conclusion:**

The present study for the first time provides a comprehensive insight into the role of product selection, optimal concentration, temperature, and cleaning duration in maximising soil removal from dental instruments.

## Introduction

The decontamination of reuseable dental instruments is a key process in infection prevention and control practice in dental clinics.^1^^,^^2^ Appropriate decontamination workflow is essential to ensure the safety of patients, staff, and students engaged in clinical dental practice.^3^ Hence, all dental clinics are required to adhere to jurisdictional infection control guidelines and regulations, and follow the guidance issued by national professional associations.^4^ Failure to follow the principles stated in these official guidelines can result in serious consequences for the patients and staff, as well as potential legal and financial repercussions.^5^

Dental instruments come into direct contact with the body fluids of patients, such as saliva and blood, and with oral tissues.^1^, ^2^, ^3^ Hence, dental instruments are exposed to a range of microorganisms, as well as biofilms in the oral cavity, and biomolecules from the patient such as proteins.^6^^,^^7^ During the dental treatment procedures, these microorganisms and biomolecules adhere to dental instruments through physical entrapment, microbial adhesion, protein adsorption and various electrostatic and hydrophobic interactions.^8^^,^^9^ Once an instrument has been used, organic material will start to dry onto the surface of the instrument.^10^^,^^11^ Bacterial products such as endotoxins may also adsorb onto the surface of instruments.^11^^,^^12^ If used instruments are left for an extended period prior to cleaning, the organic material forms stronger bonds, clumping onto the surface. At the same time, inorganic material such as calcium and phosphate salts may deposit. Overall, instruments become harder to clean if the decontamination part of the reprocessing procedure is not initiated promptly.

The reprocessing of dental instruments typically involves multiple steps to ensure they are cleaned thoroughly, and free of any contaminants, before sterilisation and reuse on further patients. The basic steps are precleaning during use and at the chairside, followed by mechanical cleaning in an ultrasonic cleaner or washer-disinfector (WD), packaging into a sterile barrier system, and then sterilisation.^1^, ^2^, ^3^ Skipping any of these steps will compromise the safety of the instruments, and increase the risk of cross-infection occurring.^13^

Items must be visibly clean before steam sterilisation since this is not a cleaning process and will not remove contaminants from dental instruments.^2^^,^^3^ As well as microorganisms, cleaning processes must remove proteins and other organic biomolecules.^14^^,^^15^ When dental instruments cannot be cleaned immediately, they need to be treated appropriately to stop substances drying onto their surfaces. This is a particular challenge at the end of the day for the instruments used on the final appointments, if they are not cleaned immediately.

Suitable holding solutions can prevent the drying of contaminants onto instruments, and make later stages in the cleaning process less challenging, by breaking down bioburden. Some holding solutions also include disinfectants that prevent the overgrowth of microorganisms that would otherwise occur.^8^ The commercial products used for holding solutions and for ultrasonic cleaning of dental instruments have different chemistries and can be divided into enzymatic-based products and nonenzymatic products.^16^ In some cases, the same product is used in an ultrasonic cleaner as well as in a holding solution.^17^^,^^18^ During ultrasonic cleaning, high-frequency sound waves generate cavitation. As microscopic bubbles collapse, the resulting fluid movements dislodge contaminants from instrument surfaces.^19^

The efficiency of ultrasonic cleaning solutions can be influenced by various parameters, such as the product concentration, the water temperature, contact time, the extent of bioburden and the complexity of the dental instruments.^20^ Understanding the influence of these parameters is crucial for maximising the efficacy of ultrasonic cleaning. Moreover, with the widespread use of ultrasonic cleaners in dental clinics worldwide, there are substantial financial implications around maximising the cost-efficiency of cleaning using this approach. To date, no studies in the literature have comprehensively examined the parameters that affect the efficacy of different chemistries against organic bioburden during the ultrasonic cleaning of dental instruments. Taking this research gap into consideration, in the present study we comprehensively evaluated the cleaning efficacy of 13 commonly used commercially available products against organic bioburden on dental instruments.

## Methods and materials

### Commercially available disinfection products

The products used in the present study are summarised in Table. The information listed on composition is based on publicly disclosed information on product labels and safety data sheets.

**Table:** The products used in this study are summarized in the table, with composition information based on publicly available details from product labels and safety data sheets.

Code	Product name and manufacturer	Enzymes	Other ingredients	Recommended concentration
A	Matrix Biofilm Remover (Whiteley Corp., Newcastle, NSW, Australia)	None	Not disclosed	10-50 mL/L
B	UC30 (Coltene Whaledent Inc., Cuyahoga Falls, OH, USA)	None	Disodium tetraborate, alkylpolyglycosides, propylene glycol	16 mL/L
C	UC32 (Coltene Whaledent Inc., Cuyahoga Falls, OH, USA)	Enzyme (not disclosed)	Disodium tetraborate, alkylpolyglycosides, propylene glycol	8 mL/L
D	Clinimax (Whiteley Corp., Newcastle, NSW, Australia)	None	Didecyldimethylammonium chloride	4-6 mL/l
E	Sonidet (Whiteley Corp., Newcastle, NSW, Australia)	None	None disclosed	5 mL/L
F	Tethyclean (Cefla SC, Imola, Italy)	None	Benzisothiazolin-3-one, amphoteric surfactants, phosphonates, sodium hydroxide, propylene glycol	5-10 mL/L
G	Neodisher Mediclean Forte (Chemische Fabrik Dr. Weigert GmbH & Co., Hamburg, Germany)	Enzyme (not disclosed)	Fatty alcohols, alkoxylated 2,2′,2″-nitrilotriethanol	2-10 mL/L
H	Asepti Multizyme (Ecolab, Macquarie Park, NSW, Australia)	Multiple enzymes	Disodium tetraborate	5-10 mL/L for manual; 2-8 mL/L for ultrasonic
I	Medizyme (Whiteley Corp., Newcastle, NSW, Australia)	Alpha-amylase (bacterial), protease	None stated	6 mL/L
J	Clinidet (Quality Medical Innovations, Heathwood, QLD, Australia)	None	Alcohol alkoxylate	4 mL/L
K	Getinge Clean Manual Pro+ (Quadralene Ltd, Derby, UK)	Subtilisins, lipase, amylase, cellulase	Nonionic surfactant, 1,2-benzisothiazol-3(2H)-one	2-20 mL/L
L	Getinge Clean Enzymatic Plus (Quadralene Ltd, Derby, UK)	Subtilisins, lipase, amylase	Nonionic surfactant, 1,2-benzisothiazol-3(2H)-one	2-20 mL/l
M	Optizyme Ultra D (Henry Schein Inc NSW, Australia)	Protease, lipase, endocellulase, alpha-amylase	Boric acid, ethoxylated alcohol, benzalkonium chloride	6 mL/L

### Testing under static (nonagitation) conditions

Albert Browne STF Load Check Indicators (STERIS Corp) were used. This indicator contains two protein sources, as well as lipids and polysaccharides. It is designed to mimic the cleaning efficacy soil test for surgical instruments in ISO/TS 15883-5:2021 (WD Part 5: Performance requirements and test method criteria for demonstrating cleaning efficacy). This indicator is ideal for testing cleaning efficiency for dental instruments using ultrasonic cleaning as it provides a very robust challenge in terms of dry organic bioburden.

First, we examined the static (nonagitated) cleaning efficiency of various products at their maximum recommended concentrations in distilled water at 35°C over a 10-minute period. For this purpose, containers with 200 mL of distilled water were immersed in a water bath at 35°C and allowed to equilibrate up to 30 minutes. Thereafter, test products were added to each container at the maximum concentration recommended by the manufacturer. The dilutions ranged from 50 mL/L (Matrix Biofilm) to 4 mL/L (Clinidet). The STF Load Check Indicator was then introduced into each container and kept for a 10-minute contact time. The indicator strips were photographed before and after the treatment.

For the next step, we selected the concentration of the most efficient product for the removal of organic material on the Browne soil test. Other products were then tested at the same dilution ratio. Strips were photographed in the same manner as previously.

### Effect of products used in ultrasonic cleaning on test soil

In the second set of experiments, we examined the efficacy of test products at their highest concentration when used in an ultrasonic cleaner with Browne STF Load Check Indicators, at both room temperature (25°C) and under warm conditions (40°C). Given the potential impact of water hardness on performance, tap water (208 ppm measured by Model COM-100, HM Digital, Inc.) was used. The effect of exposure time on cleaning efficiency was assessed at 1, 5, and 10 minutes.

### Effect of product concentration

In the next set of experiments, the effect of different products when used at their minimum recommended concentration was evaluated, using Browne STF Load Check Indicators. The test strips were immersed in an ultrasonic cleaner, with the temperature set to either room temperature (25°C) or warm conditions (40°C), for exposure times of 1, 5 or 10 minutes. This approach was used to assess how well using the minimum concentration of test products in ultrasonic cleaners can provide cost-effective and efficient cleaning.

Thereafter, we repeated the same experiments as described above, but this time using the concentration of each product that produced the best outcome in terms of cleaning.

### Cleaning of artificially soiled dental instruments

For this set of experiments, we artificially soiled a set of 10 dental instruments, including tweezers and scalers, using a commercially available standard test soil for washer-disinfectors (Steris Albert Browne Edinburgh Test Soil), which conforms to TS/ISO 15883-5. The dental instruments were coated with the Edinburgh test soil using a brush, and allowed to air dry for 60 minutes.^21^ Thereafter, the instruments were exposed to the test products at their maximum recommended concentrations in the ultrasonic cleaner for 30 minutes.

The efficacy of cleaning was evaluated by ProReveal technology (Synoptics Health) following the manufacturer's instructions. Each dental instrument was placed on matte nonreflective black paper and sprayed with the ProReveal fluorescence reagent and then imaged to show residues of protein.^21^^,^^22^ Lower fluorescence values after ultrasonic cleaning represent lower levels of residual protein from Edinburgh soil, and thus higher cleaning efficacy. Data for protein quantification were used to determine the cleaning ‘status’ of the analysed instruments. The study followed the guidelines set of ISO 15883-5, which specifies an upper limit of 6.4 μg/cm^2^ of residual protein on the instrument as the acceptable threshold for contamination after processing. The limit of detection for the method used was 50 ng protein/cm^2^ instrument surface.

For the final set of experiments, the test products were used in a similar manner, but with a shorter exposure time (10 minutes), and then assessed the efficacy of cleaning dental instruments was using ProReveal.

## Results

### Cleaning efficiency of test products on organic bioburden under static conditions

**Fig. 1:**
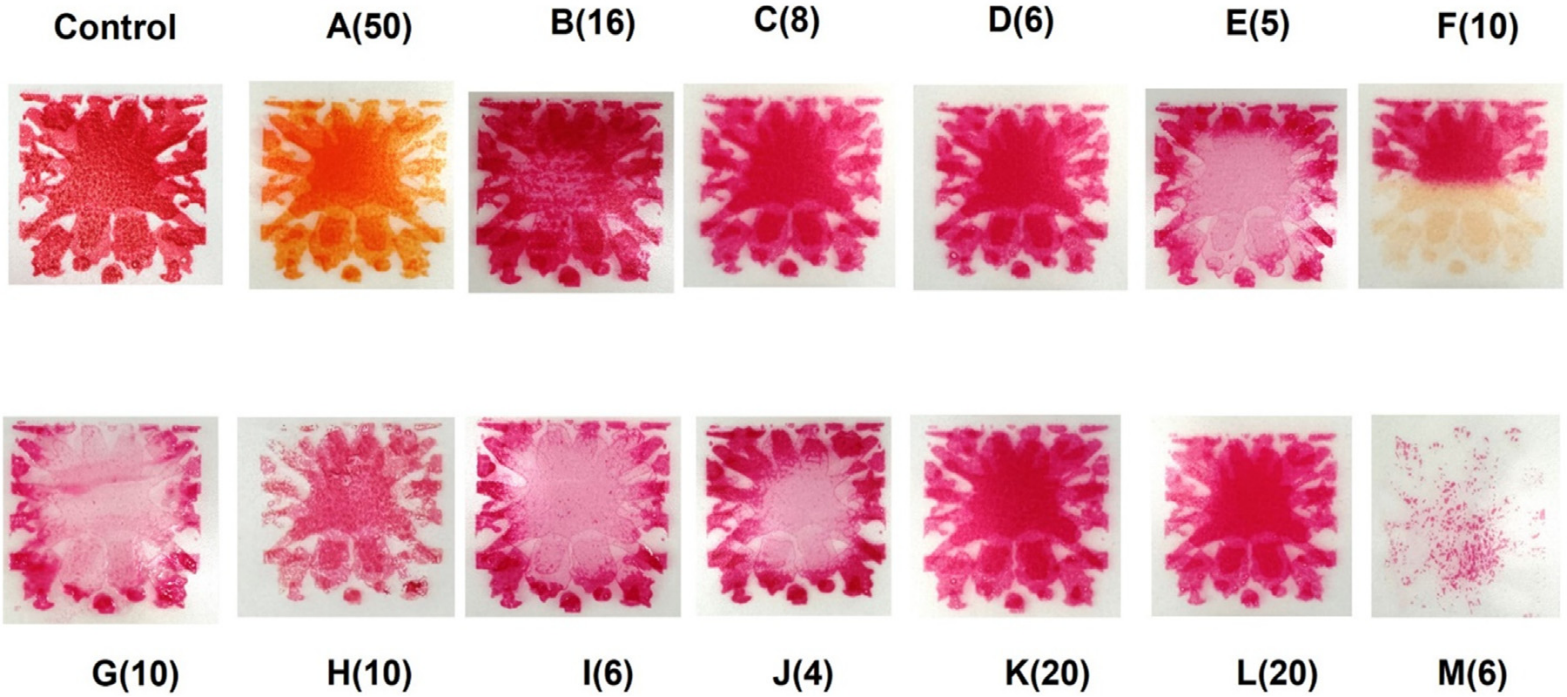
Static cleaning efficacy for products at maximum manufacturer-recommended concentrations in distilled water at 35°C for 10 minutes using STF Browne Load Check indicators. The letters indicate the codes of the products shown in Table 1 (number within brackets indicates the concentration used in mL/L).

The data for performance when products were used at their maximum recommended concentrations, in static (nonagitated) conditions, is summarised in Figure 1. There were obvious differences in performance among the test products for the robust challenge of the Browne STF Load Check Indicators. Superior cleaning efficacy is demonstrated by reduced staining on the STF Browne Load Check indicators, as this reflects a higher degree of soil removal and, consequently, more effective cleaning performance. The best-performing product was Optizyme Ultra D (6 mL/L, labelled ‘M’ in Figure 1) which consistently delivered superior cleaning results, with Asepti Multizyme (labelled H) showing moderate efficacy. Other tested products were ineffective in the static cleaning mode.

In the next experimental series, all products were tested at the same dilution as product M (Optizyme Ultra D at 6 mL/L) under the same experimental conditions (Supplementary Figure 1). In this series, Optizyme Ultra D consistently achieved the most thorough removal of the test soil, outperforming all other products.

### Efficacy of products when used in ultrasonic cleaning

**Fig. 2:**
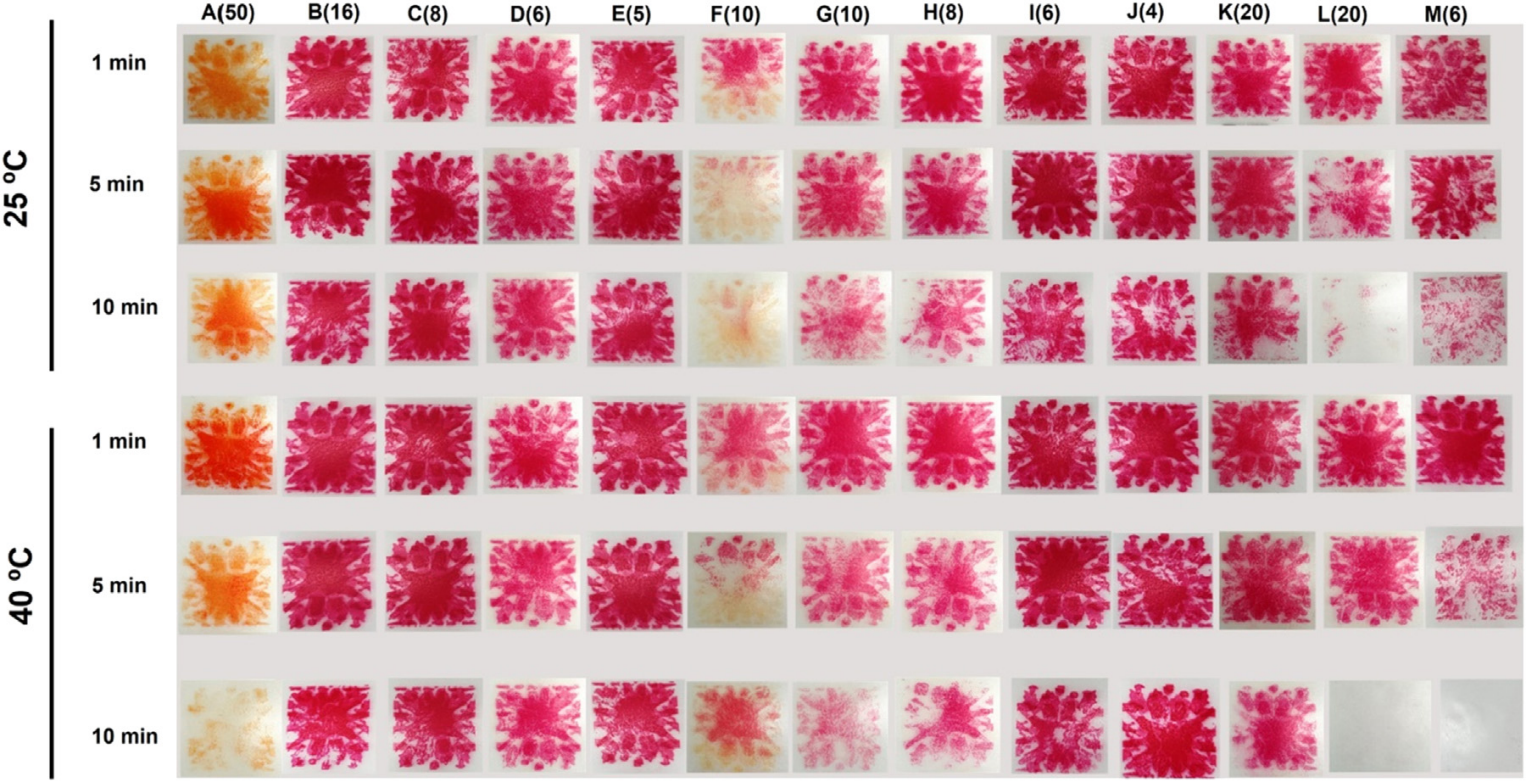
Effect of temperature and time on ultrasonic cleaning efficacy for products at maximum manufacturer-recommended concentrations in tap water (number within brackets indicates the concentration used in mL/L).

All products, when used at their highest recommended concentrations, showed greater effectiveness for Browne STF Load Check Indicators when used in an ultrasonic cleaning cycle than under static conditions with no agitation (Figure 2).

Highly effective products that removed material from test strips were Mediclean Forte (10 mL/L), Asepti Multizyme (8 mL/L), Getinge Enzymatic Plus (20 mL/L), and Optizyme Ultra D (6 mL/L). On the other hand, there was little removal of material from test strips for UC30 (16 mL/L), UC32 (8 mL/L), Clinimax (6 mL/L), Sonidet (5 mL/L), Medizyme (6 mL/L), and Clinidet (4 mL/L). Matrix (50 mL/L) changed the colour of the test strips and was moderately effective.

For the products that were very effective, there was superior cleaning performance at 40°C than at 25°C, and at an exposure time of 10 minutes rather than at shorter intervals (Figure 2). Overall, the greatest visible reduction in the test soil was achieved at 40°C for 10 minutes using Getinge Enzymatic Plus (20 mL/L) and Optizyme Ultra D (6 mL/L).

In the next set of experiments, all products were tested at the minimum concentration recommended by the manufacturer. Under these conditions, only one product (Optizyme Ultra D at 6 mL/L) was effective in cleaning the organic bioburden from the test strips (Supplementary Figure 2).

**Fig. 3:**
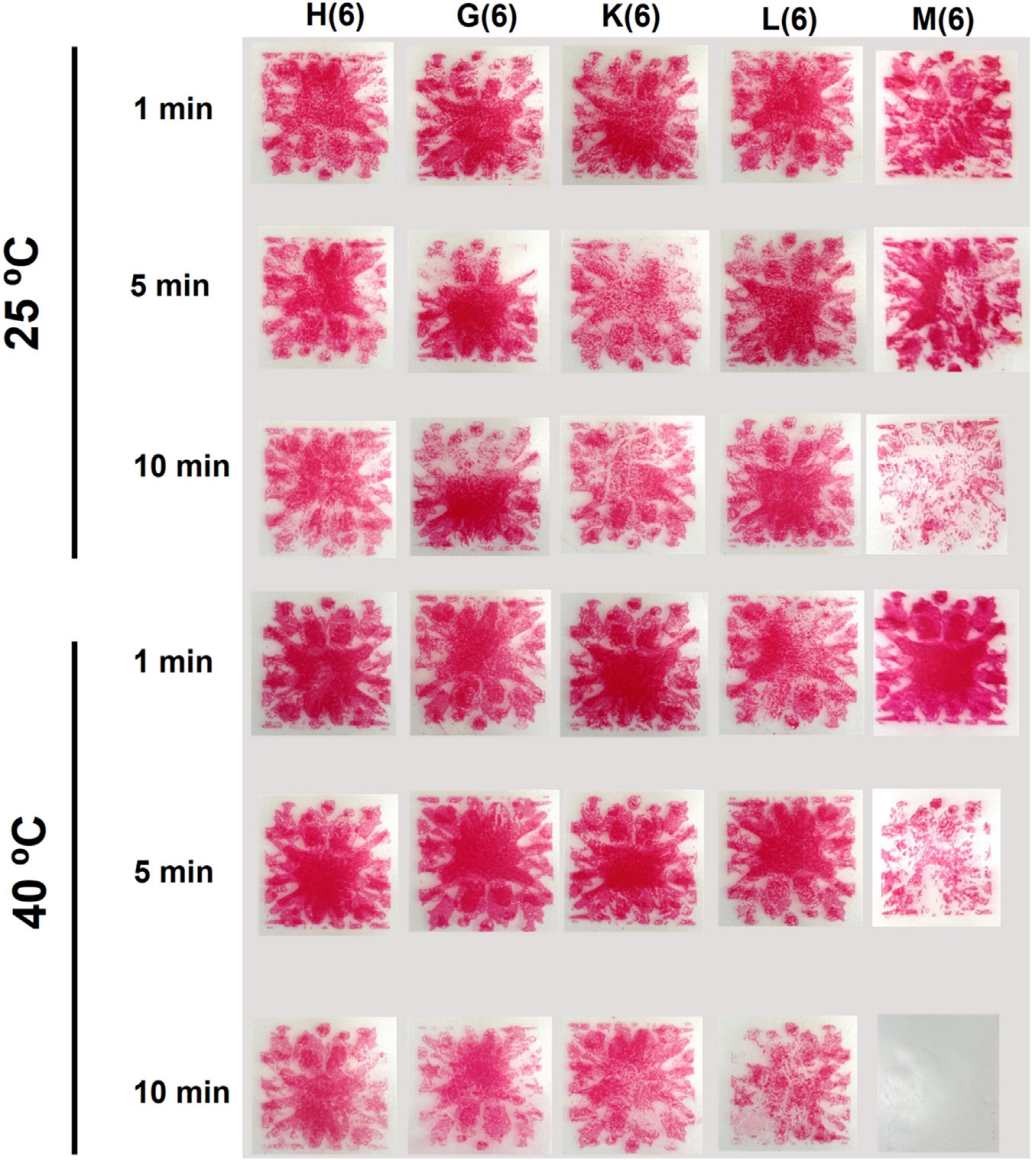
Effect of temperature and time on ultrasonic cleaning efficacy for products used at 6 mL/L in tap water (number within brackets indicates the concentration used in mL/L).

When the selected products which showed efficacy at high concentrations were tested at a dilution of 6 mL/L, only Optizyme Ultra D was effective on the test strips. The test soil was removed completely by this product in 10 minutes at 40°C (Figure 3).

### Efficacy of test products in ultrasonic cleaning of artificially soiled dental instruments

#### Long ultrasonic cleaning cycle

**Fig. 4:**
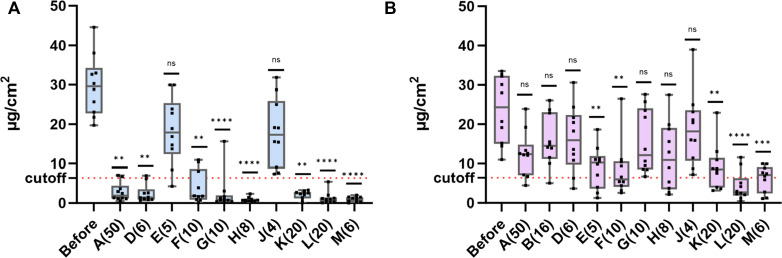
(A) Quantitative analysis of residues on artificially soiled dental instruments using PROReveal for a long ultrasonic cleaning cycle of 30 minutes for products at maximum manufacturer-recommended concentrations in tap water. Statistical analysis was performed using Kruskal–Wallis test and is indicated as *P < .05, **P < .01, ***P < .001, ****P < .0001, and ‘ns’ for not significant (number within brackets indicates the concentration used in mL/L). (B) Quantitative analysis of residues on artificially soiled dental instruments using PROReveal for a short ultrasonic cleaning cycle of 10 minutes for products at maximum manufacturer-recommended concentrations in tap water. Statistical analysis was performed using Kruskal–Wallis test and is indicated as *P < .05, **P < .01, ***P < .001, ****P < .0001, and ‘ns’ for not significant (number within brackets indicates the concentration used in mL/L).

In this part of the study, the cleaning efficacy of various products when used at their highest recommended concentrations in ultrasonic cleaners was evaluated using artificially soiled dental instruments and a 30-minute cleaning cycle. Selected products that had performed well in previous tests were chosen for additional analysis. The quantitative analysis of residual contamination on instrument surfaces postcleaning, measured by fluorescence intensity using the PROReveal method ^21^^,^^22^ is shown in Figure 4A. The best-performing products were Optizyme Ultra D (6 mL/L), Asepti Multizyme (8 mL/L), and Getinge Enzymatic Plus (20 mL/L), as these showed the least residual protein. Moderate performance was found for Mediclean Forte (10 mL/L), Clinimax (6 mL/L), Matrix Biofilm Remover (50 mL/L), and Getinge Manual Pro+ (20 mL/L). The lowest level of performance was seen for Tethyclean (10 mL/L), Sonidet (5 mL/L), and Clinidet (4 mL/L). Images of the residual protein after ultrasonic cleaning are shown in Supplementary Figure 3.

#### Short ultrasonic cleaning cycle

Data for residual protein remaining after a 10-minute ultrasonic cleaning cycle are presented in Figure 4B, using the PROReveal method. The lowest fluorescence signal from residual protein was seen for Optizyme Ultra D (6 mL/L) and Getinge Enzymatic+ (20 mL/L). Their overall cleaning performance in this 10-minute cycle was similar to that seen at 30 minutes.

**Fig. 5:**
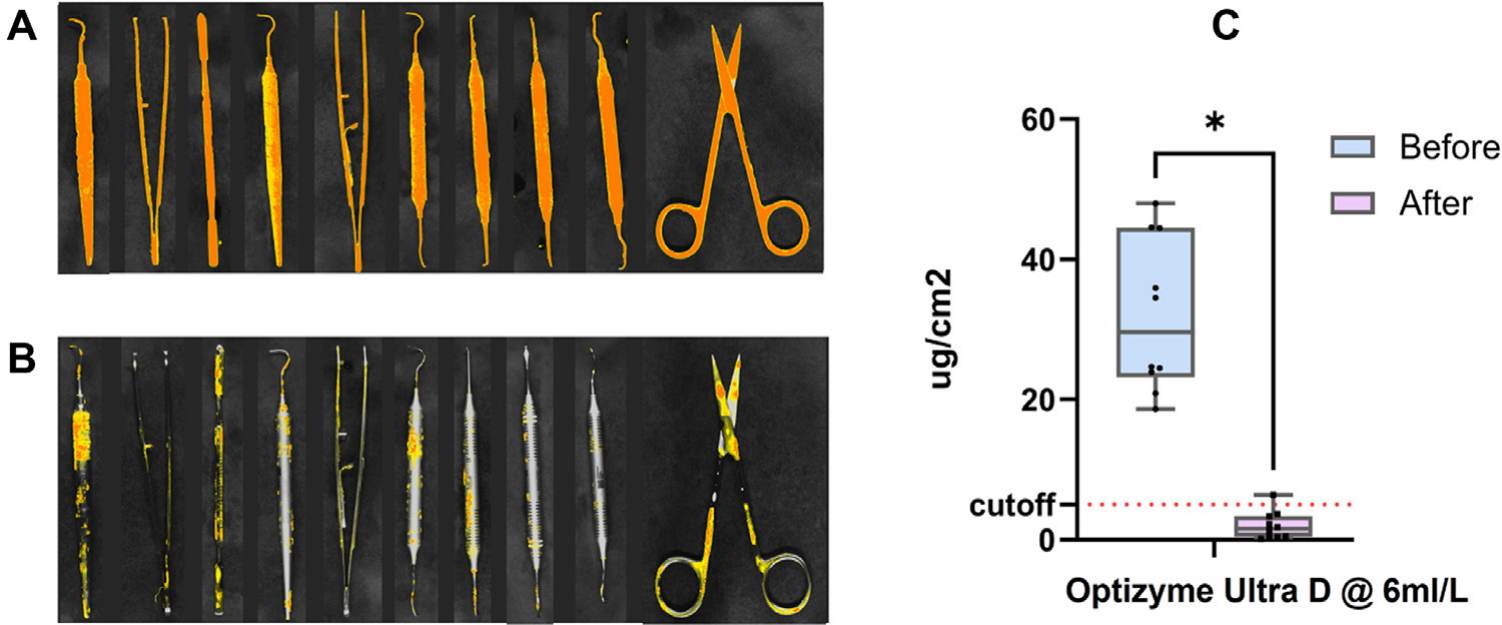
Visual and quantitative analysis of residues on artificially soiled dental instruments using PROReveal for a long ultrasonic cleaning cycle of 30 minutes using product M. The instruments were dried for 3 days before cleaning. Panel (A) shows the instruments immediately before cleaning; while panel (B) shows the instruments after cleaning. Panel (C) compares the levels before and after.

On the other hand, other products showed reduced performance with the shortened cycle. Matrix Biofilm Remover (50 mL/L), Tethyclean (10 mL/L), and Getinge Manual Pro+ (20 mL/L), provided moderate contaminant removal in 10 minutes, albeit with reduced efficiency compared to the longer 30-minute cycle. The remaining products showed high levels of residue and greater variability. An example of residual protein is shown in Supplementary Figure 4.

### Efficacy of an enzymatic-based product for ultrasonic cleaning of dried organic bioburden on dental instruments

Optizyme Ultra D detergent was subjected to an additional, in-depth analysis in the long ultrasonic cleaning cycle to assess cleaning of instruments that were soiled and then kept for 3 days for drying prior to cleaning. Ultrasonic cleaning gave a substantial reduction in residual protein, with quantitative measures showing a marked decrease for the 30-minute ultrasonic cycle (Figure 5).

## Discussion

The results of the current study provide multiple insights into the performance of agents used for ultrasonic cleaning, with the variables of product choice, dilution ratio, temperature, and cleaning time being important factors that influence the removal of bioburden. Despite the critical importance of effective cleaning and reprocessing in dentistry,^1^^,^^2^^,^^10^ there is limited published data on comparative performance of products used in ultrasonic cleaners.^21^^,^^23^ In the present study, we evaluated the comparative efficacy of commercially available products using ordinary tap water (208 ppm), reflecting real-world conditions.^24^^,^^25^

The first part of the study demonstrated that under static conditions only Optizyme Ultra D (6 mL/L) was effective in cleaning bioburden. The capability to remove organic bioburden without the need for ultrasonic agitation is relevant in clinical setting as a holding solution where instruments are kept while awaiting cleaning. The present results also show the impact of product dilution ratios on cleaning performance and reinforce previous conclusions that the concentration of products significantly affects cleaning efficacy.^26^ When tested at similar dilution ratios, Optizyme Ultra D at 6 mL/L was superior to other products for ultrasonic cleaning.

The inclusion of cleaning additives is essential for effective ultrasonic cleaning.^17^^,^^18^ The findings of this study demonstrate that the choice of product has a powerful influence on the removal of organic bioburden. Ultrasonic cleaning is used widely in dental clinics because it is effective on rigid solid dental instruments and superior to manual cleaning.^18^^,^^19^ Overall, the best-performing additives for ultrasonic cleaning were Mediclean Forte (10 mL/L), Asepti Multizyme (8 mL/L), Getinge Enzymatic Plus (20 mL/L), and Optizyme Ultra D (6 mL/L). All four were highly effective in removing the test soil from Albert Browne STF Load Check Indicators.

It is essential to follow the manufacturer's instructions regarding the temperature of the water used in ultrasonic cleaning.^4^ The present results show that cleaning efficacy was influenced by water temperature, with superior performance at 40°C compared to 25°C. Higher temperatures not only enhance the cavitation effect, but also improve the solubility and reactivity of cleaning agents, leading to superior cleaning results.^19^ Overall, Getinge Enzymatic Plus (20 mL/L) and Optizyme Ultra D (6 mL/L) were the most effective products at 40°C, and both are enzyme-based. This reinforces the need to ensure the optimum temperature is used for enzyme-based products.^27^ Interestingly, when the minimum recommended concentration of the commercial products was used, only Optizyme Ultra D, which has a single stated use concentration (6 mL/L), was effective in removing the test soil from the STF Load Check Indicator strips.

To assess protein residues on dental instruments, the present study used Edinburgh Test Soil to artificially soil a range of dental instruments, prior to ultrasonic cleaning in 10- and 30-minute cycles, with products used at their highest recommended concentrations. There was considerable variability in performance, with the enzymatic products providing superior cleaning results. It was notable that two enzymatic products L (Optizyme Ultra D (6 mL/L)) and M (Getinge Enzymatic Plus (20 mL/L)) demonstrated comparable efficacy in 10 and 30 minute cycles, while other products showed much better performance in the longer cycles. The results showed no statistical difference in results between L and M even though Product ‘L’ is dosed 3.33 times higher than sample ‘M’.

The time required for effective cleaning forms a key part of the overall reprocessing sequence. Longer cleaning times reduce the availability of instruments for use in the clinic and require the clinic to have a larger inventory of instruments. This is a further cost impact to the dental clinic and adds to the effects of product cost and product dilution ratios. While there may be savings with greater dilution of products, the longer cleaning time required makes this counterproductive.

The final part of the study tested a worst-case scenario where soil was dried onto dental instruments for 3 days. This was designed to replicate the situation where items were left over a weekend. Since enzyme-based products were found to be most effective, we used Optizyme Ultra D in a 30-minute cycle. This was able to reduce the organic bioburden on the instruments. Despite this, a better approach to leaving instruments dry over a weekend period would be to immerse them in a holding solution with potent antimicrobial activity, to prevent microbial growth.

The present study has several limitations that must be acknowledged. The challenge posed to cleaning was very high since the test strips and Edinburgh soil were designed for WD. Using these provided a very difficult but consistent level of challenge, making this suitable for exploring variables of interest. Further studies should be conducted using instruments from working dental clinics, especially using the fluorescence method to detect residual protein. Finally, the dental instruments used in the study were constructed of rigid stainless steel and had simple designs with limited complexity. Further studies are needed to explore ultrasonic cleaning for other instrument types.

In conclusion, the present study shows that effective ultrasonic cleaning requires the careful consideration of a range of factors, to ensure that cleaning outcomes are consistent. There were notable differences in performance among the tested products with different chemistries. The novel findings underscore the importance of selecting appropriate products, and then using them at suitable dilutions, in warm water, and for sufficient time, to maximise the efficiency of cleaning dental instruments.

## Conflict of interest

The authors declare that they have no known competing financial interests or personal relationships that could have appeared to influence the work reported in this article.

## Appendix Supplementary materials

Image, application 1

## Footnotes

Supplementary material associated with this article can be found in the online version at doi:10.1016/j.identj.2025.02.009.
