# Supplementary material for: Efficacy of Ultrasonic Cleaning Products With Various Disinfection Chemistries on Dental Instruments Contaminated With Bioburden
Source: Int Dent J. 2025 Mar 25;75(3):1632–9. doi: 10.1016/j.identj.2025.02.009 (PMC11985109; doi:10.1016/j.identj.2025.02.009)

**Supplementary Figures**

**Supplementary Fig 1.** Static cleaning efficacy for products used at a dilution of 6 ml/L in distilled water at 35 °C for 10 minutes. (Number within brackets indicates the concentration used in ml/L).


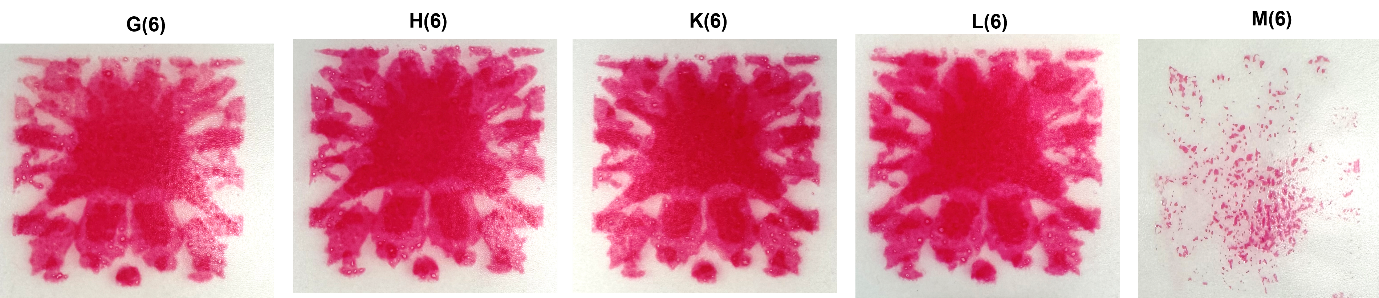


**Supplementary Fig 2.** Effect of temperature and time on ultrasonic cleaning efficacy for products at minimum manufacturer recommended concentrations in tap water. (Number within brackets indicates the concentration used in ml/L).

**
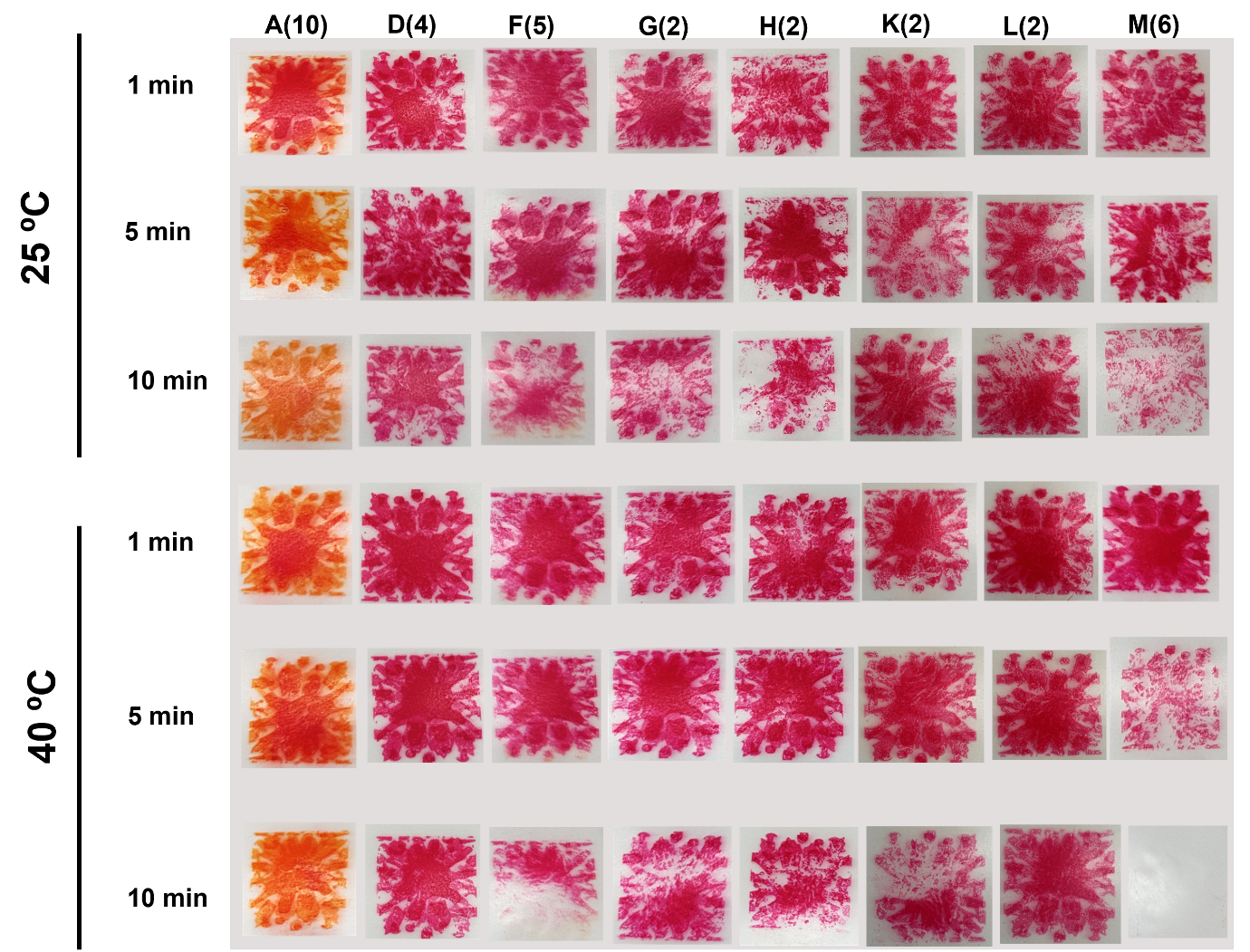
**

**Supplementary Fig 3**. PROReveal visualisation of cleaning of artificially soiled dental instruments by ultrasonic cleaning for 30 minutes. (Number within brackets indicates the concentration used in ml/L).


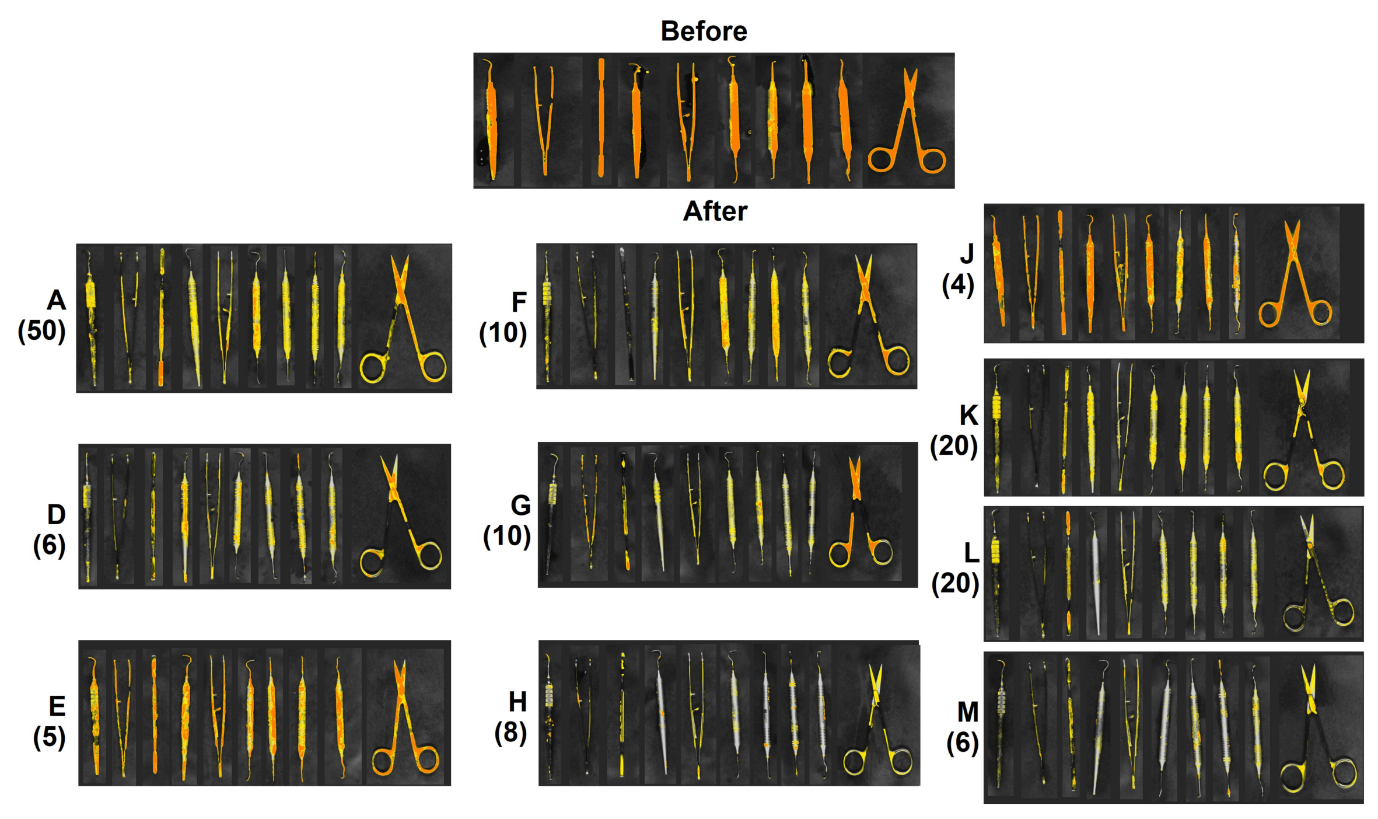


**Supplementary Fig. 4.** PROReveal visualisation of cleaning of artificially soiled dental instruments by ultrasonic cleaning for a cleaning cycle of 10 minutes. (Number within brackets indicates the concentration used in ml/L).


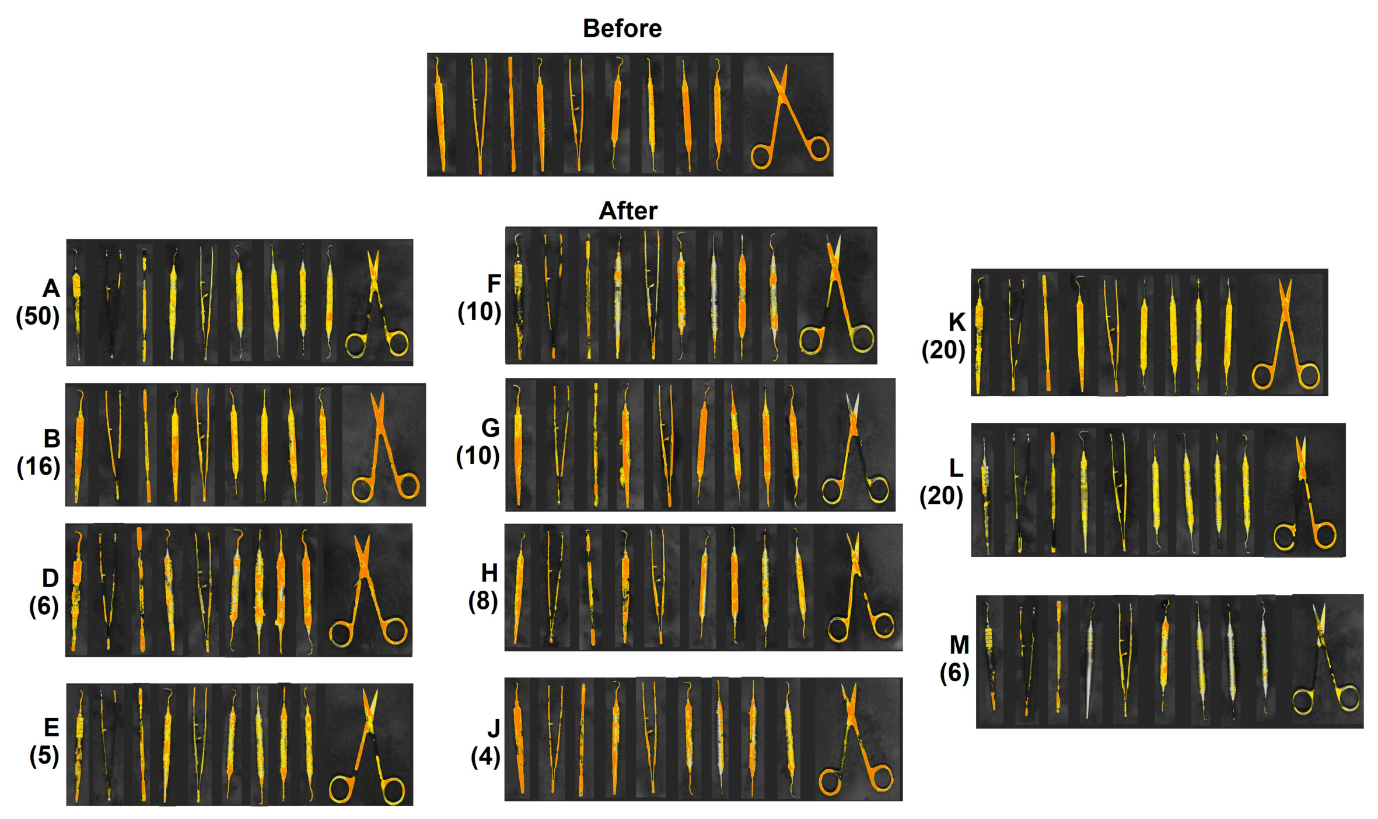

Supplement: Supplementary file 1 [file mmc1.docx]
